# Crowdsourcing with the drift diffusion model of decision making

**DOI:** 10.1038/s41598-024-61687-y

**Published:** 2024-05-17

**Authors:** Shamal Lalvani, Aggelos Katsaggelos

**Affiliations:** Department of Electrical and Computer Engineering, Northwestern University, Evanston, 60201 USA

**Keywords:** Human behaviour, Learning algorithms

## Abstract

Crowdsourcing involves the use of annotated labels with unknown reliability to estimate ground truth labels in datasets. A common task in crowdsourcing involves estimating reliabilities of annotators (such as through the sensitivities and specificities of annotators in the binary label setting). In the literature, beta or dirichlet distributions are typically imposed as priors on annotator reliability. In this study, we investigated the use of a neuroscientifically validated model of decision making, known as the drift-diffusion model, as a prior on the annotator labeling process. Two experiments were conducted on synthetically generated data with non-linear (sinusoidal) decision boundaries. Variational inference was used to predict ground truth labels and annotator related parameters. Our method performed similarly to a state-of-the-art technique (SVGPCR) in prediction of crowdsourced data labels and prediction through a crowdsourced-generated Gaussian process classifier. By relying on a neuroscientifically validated model of decision making to model annotator behavior, our technique opens the avenue of predicting neuroscientific biomarkers of annotators, expanding the scope of what may be learnt about annotators in crowdsourcing tasks.

## Introduction

Crowdsourcing involves the use of a crowd (a collection of annotators) to label datasets, and generally assumes that reliabilities of annotators in the crowd are unknown^1^. In the past few decades, crowdsourcing has proliferated to many domains, including medicine and public health^2,3^, physics and engineering^4–6^, and behavioral science.^7^ Novel applications of crowdsourcing models with machine learning involve scoring of histological and immunochemistry images^8,9^, detection of gravitational waves^10^ and flood forecasting^11^.

In crowdsourcing, an important task is to estimate annotator reliability. In the case where annotators must provide binary labels to data (e.g., 0 or 1), annotator reliability may be understood through the sensitivity and specificity of each annotator (a confusion matrix may more generally be used in the non-binary case)^12^. A common objective is to use the annotated labels to build a classifier to generalize to new data, which typically involves use of the sensitivities and specificities of the annotators^8,12^. Frequentist approaches directly estimate the sensitivities and specificities of annotators (or the confusion matrix), such as through the Expectation Maximization algorithm^12^. In the Bayesian approach, beta or dirichlet distributions are typically used as priors to the confusion matrix of the annotators^8,12,13^.

In this paper, we consider the case where annotators must provide binary labels to data in a crowdsourcing task. Rather than imposing a beta or dirichlet distribution as a prior on annotator reliability, we assume that annotators provide labels based off of a model of decision making known as the drift-diffusion model. Drift-diffusion models have been studied extensively in the context of human decision making in neuroscience and psychology^14,15^. Drift-diffusion models apply to two-choice decisions, where the decision maker most make a decision between two options, typically in a matter of seconds^16,17^. Drift-diffusion models have popularized in neuroscience due to studies showing that drift-diffusion parameters have neurological correlates with brain regions such as the prefrontal cortex, subthalamic nucleus and basal ganglia^16,18^, and correlate to dopaminergic activity in perceuptual decision making^19^.

To the knowledge of the authors, no related work considers psychological or neuroscientifically validated models as priors in crowdsourcing tasks; however, there is novel and emerging (albeit nascent) literature merging crowdsourcing in the context of neuroscience and psychology^20^. In previous literature, functional near-infrared spectroscopy (fNIRS) in conjunction with brain-computer interface (BCI) integrates neuroscience into crowdsourcing tasks^21^. Crowdsourcing tasks have also been used to collect psychological and neurological properties of annotators, which allow for determination of behavioral factors of the annotators. One study implemented an alcohol purchase task via crowdsourcing platform Amazon Mechanical Turk to identify behavioral economic factors of alochol misuse^22^. Other examples further include possibilities for cross-sectional designs for addiction science^23,24^ and even other health related challenges^25,26^, including autism^27^. Novel literature has also studied synchrony related metrics via brain coupling between individuals^28^. Finally, as may be inferred from large language models such as ChatGPT, the advent of large crowdsourced datasets have allowed advances in psycholinguistics and semantic models (see^29^ for a review).

**Figure 1:**
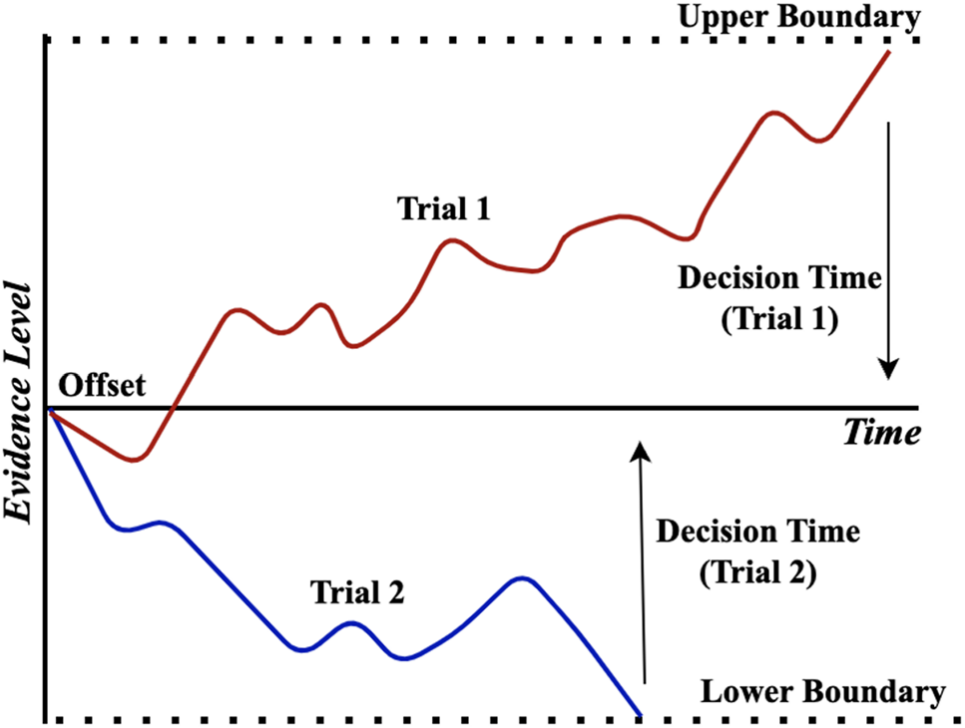
The drift-diffusion model of decision making in a two-choice task. Two hypothetical decision-making trials are displayed (Trial 1 and Trial 2). For each trial, the evidence level starts off at the offset. The evidence level evolves throughout time according to a drift diffusion process. The process terminates when the evidence level reaches either the upper boundary or lower boundary, after which a decision is made. The time for the process to reach reach the upper or lower boundary is known as the decision time. Termination of the process at the upper boundary generally corresponds to a correct decision being made. Termination of the process at the lower boundary generally corresponds to an incorrect decision being made.

**Figure 2:**
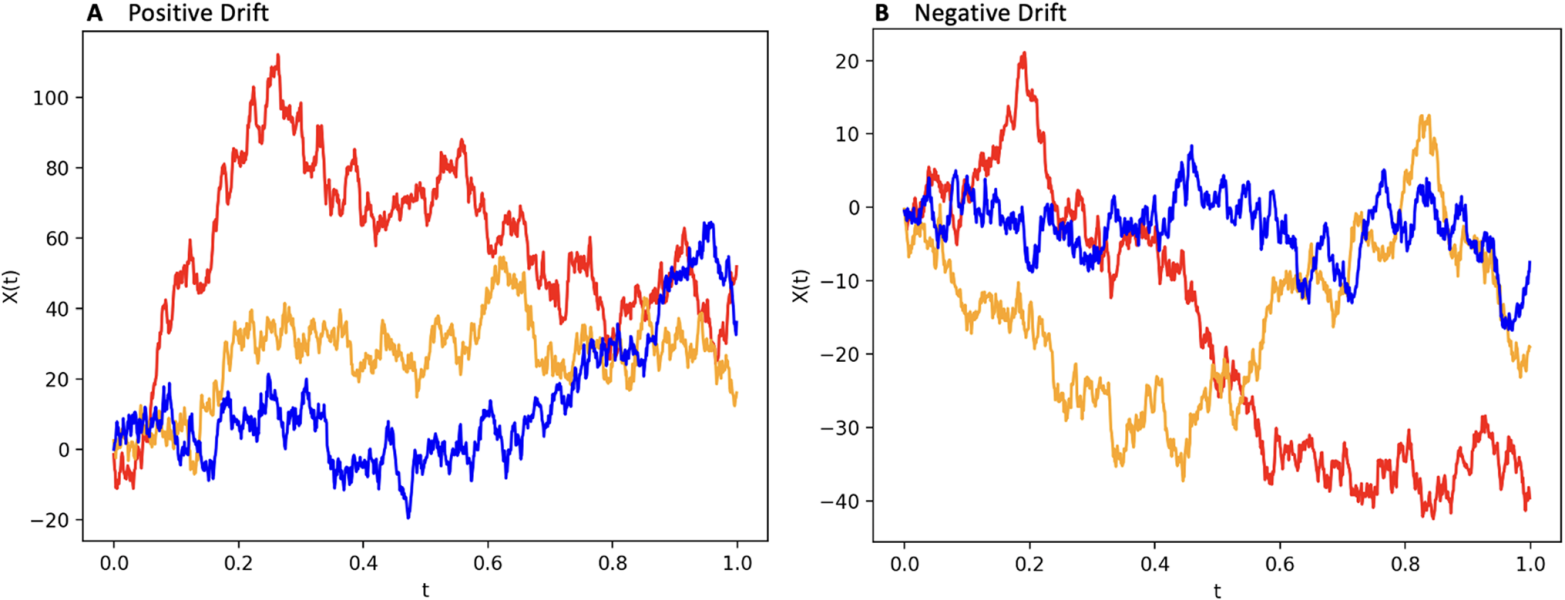
Sample paths of a drift diffusion process with positive and negative drift (which bias the processes upwards and downwards respectively), simulated with 1000 steps and a time spacing of 0.001 seconds. (**A**) Three random sample paths are simulated of a drift diffusion process with drift rate of.01 and diffusion rate of 2. (**B**) Three random sample paths are simulated of a drift diffusion process with drift rate of $-0.01$ and diffusion rate of 1.

Drift-diffusion models are illustrated as follows. Consider a decision making task, where the decision maker must make a decision between two options. In drift-diffusion models, it is assumed that the decision making process may be modeled by a latent stochastic process. This process is informally known as the ’evidence level’. Intuitively, the value of this process at any given time is interpreted as the amount of evidence the decision maker has obtained in making their decision. Further, it is assumed that this process evolves throughout time according to a drift-diffusion process, and terminates when the process reaches either an upper boundary or lower boundary. A common convention is that termination at the upper and lower boundaries respectively correspond to correct and incorrect decisions being made (Fig. 1). The evidence level, which evolves according to a drift-diffusion process, is specified by the drift rate ($\mu$) and the diffusion rate ($\sigma$). The evidence level (denoted *X*(*t*), where *t* is time) evolves in a time period *dt* according to the equation below (Fig. 2):

1 $$\begin{aligned} X(t) = \mu dt + \sigma dW(t), \end{aligned}$$

where *W*(*t*) is a Wiener Process (i.e. *W*(*t*) is normally distributed with mean zero and variance *t*).

This paper presents a framework for the usage of drift-diffusion models in a crowdsourcing task as a prior on annotator decision making. Specifically, it is assumed that annotators in a binary labeling task produce labels according to drift-diffusion processes that depend on the ground truth labels. Variational inference is used to obtain estimates on annotator parameters (such as the drift and diffusion rates), as well as estimates of ground-truth labels. The technique additionally allows for construction of a Gaussian process classifier, so that labels may be predicted from data not labeled by annotators. While the binary labeling case is considered as the first step for this new framework (such as in seminal work work in crowdsourcing^12^), the analysis of the non-binary label case is outside the scope of this study and considered an item for future work (see *discussion* for notes on the computational complexity in the non-binary case and additional future work).

## Mathematical description of crowdsourcing problem

**Figure 3:**
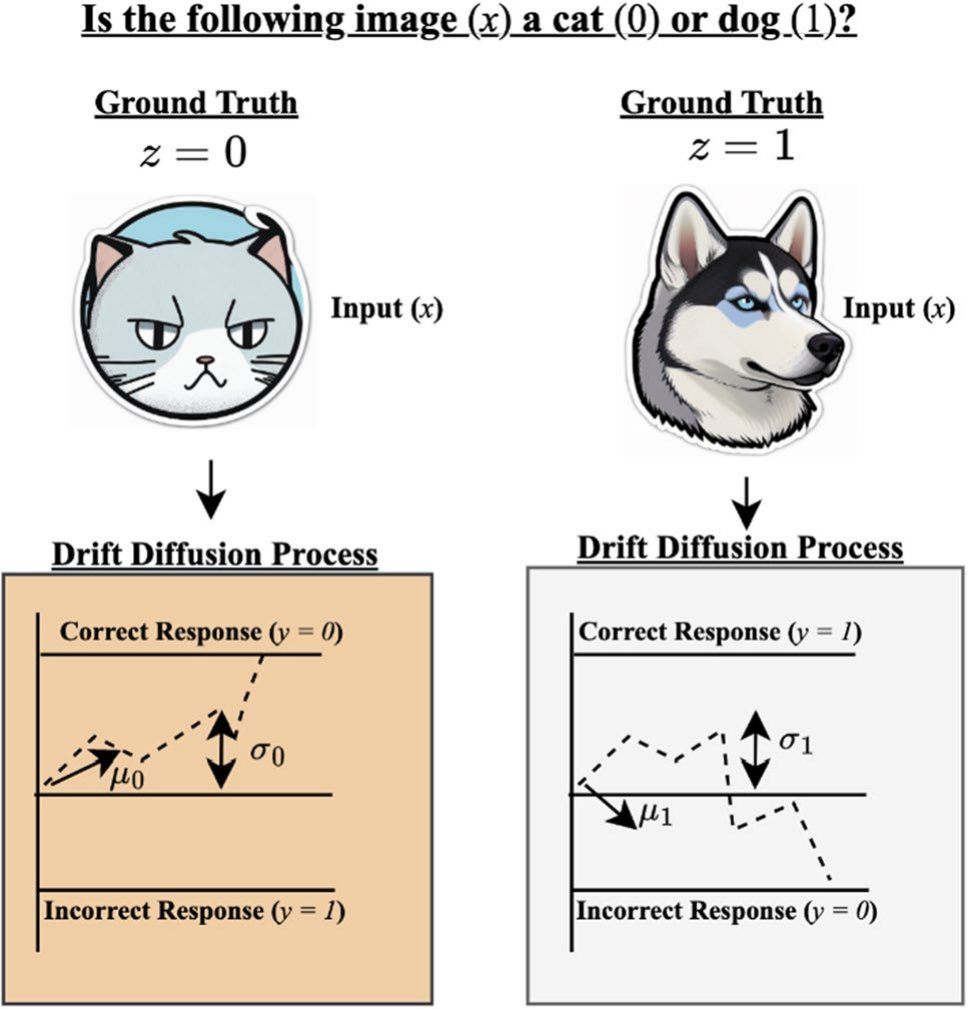
Generation of annotator labels. For simplicity, superscripts and subscripts identifying the annotator, input and ground truth are dropped. An image *x* is crowdsourced to an annotator to be labeled as a cat (label y = 0) or dog (label y = 1). Conditional on the ground truth *z* of image *x*, the annotator generates a label according to drift diffusion process. If the image is a cat ($z = 0$), a drift-diffusion process occurs with drift and diffusion rates $\mu _0$ and $\sigma _0$ respectively, with a correct label ($y=z=0$) being generated if the process hits the upper boundary. If the image is a dog ($z=1$), a drift-diffusion process occurs with drift and diffusion rates $\mu _1$ and $\sigma _1$ respectively, with a correct label ($y=z=1$) being generated if the process hits the upper boundary. The drift and diffusion rates are independent of the input, conditional on the ground truth.

Consider a set of inputs $x_i \in \mathbb {R}^m$ ($1 \le i \le n$, $m \ge 1$) with unknown binary ground truth labels $z_i$ (i.e. $z_i = 0$ or $z_i = 1$). Let there be *k* annotators who annotate each input $x_i$, providing a binary label $y^j_i$, where *j* denotes the *j*-th annotator ($1 \le j \le k$). Drift-diffusion models generally provide parameters *specific* to a task. It is therefore assumed that conditional on the ground truth label (e.g., a ground truth recognition task), annotators produce labels according to a drift-diffusion process (Figure 3), which in particular is conditionally independent of the input. Assume that the offset, upper boundary and lower boundary for all annotators are given by 0, +1 and −1, so that the drift and diffusion parameters are the only unknown parameters of the annotators. Let $\mu ^j_0$ and $\sigma ^j_0$ denote the respective drift and diffusion parameters of annotator *j* conditional on $z = 0$, and similarly, let $\mu ^j_1$ and $\sigma ^j_1$ denote the respective drift and diffusion parameters of annotator *j* conditional on $z = 1$. The following objectives are considered in the manuscript.

1. Determine the drift-diffusion parameters $\mu ^j_0,\sigma ^j_0,\mu ^j_1,\sigma ^j_1$ for every annotator *j*.
2. Determine the sensitivity $P(y^j = 1|z^j=1)$ and specificity $P(y^j=0|z^j=0)$ for each annotator *j*.
3. For each input $x_i$, determine a probabilistic label $p_i = P(z_i = 0)$.
4. Build a classifier $f:\mathbb {R}^m \rightarrow \{0,1\}$ that assigns a label $z=f(x)$ for any input *x*, where *x* need not be in the crowdsourced dataset.

## Bayesian implementation

Following similar approaches^12,13^, the log-likelihood of the observed data is shown below. Subsequently, the log likelihood is used for variational inference.

### Derivation of log likelihood

For ease of notation, let $\textbf{Y}_i = \{y_i^j\}_{j=1}^k$, $\textbf{Y} = \{\textbf{Y}_i \}_{i=1}^n$ and $\textbf{X} = \{x_i\}_{i=1}^n$. Define $\varvec{\mu }_0 = \{ \mu _0^j \}_{j=1}^k$, $\varvec{\mu }_1 = \{ \mu _1^j \}_{j=1}^k$, $\varvec{\sigma }_0 = \{ \sigma _0^j \}_{j=1}^k$, and $\varvec{\sigma }_1 = \{ \sigma _1^j \}_{j=1}^k$. Let $\textbf{p} = \{p_i\}_{i=1}^n$, where $p_i = \mathbb {P}(z_i=1)$. Note that by application the optional stopping theorem for a bounded drift diffusion process (see Ross (1996)^30^), the sensitivities and specificities for annotator *j* may be obtained conditional on the drift and diffusion parameters (see Supplemental Information) as follows, where *S* denotes the logistic function (e.g., $S(a) = \frac{1}{1+e^{-a}}$).

2 $$\begin{aligned} P_j\left( y=0|z=0,\mu _0^j,\sigma _0^j\right) = S\left( \frac{2\mu _0^j}{(\sigma _0^j)^2}\right) \end{aligned}$$

3 $$\begin{aligned} P_j\left( y=1|z=1,\mu _1^j,\sigma _1^j\right) = S\left( \frac{2\mu _1^j}{(\sigma _1^j)^2}\right) \end{aligned}$$

The log-likelihood may be derived as (see Supplemental Information),

4 $$\begin{aligned}{} & {} ln \textit{ } P(\textbf{Y}|\varvec{\mu }_0,\varvec{\mu }_1,\varvec{\sigma }_0,\varvec{\sigma }_1,\textbf{p}) = \sum _{i=1}^n ln \textit{ } \left( \sum _{j=1}^k p_i \left( 1-S\left( \frac{2\mu _0^j)}{(\sigma _0^j)^2}\right) \right) ^{y_i^j} \left( S\left( \frac{2\mu _0^j}{((\sigma _0^j)^2}\right) \right) ^{1-y_i^j}\right. \nonumber \\{} & {} \quad \left. + (1-p_i) \left( S\left( \frac{2\mu _1^j}{(\sigma _1^j)^2}\right) \right) ^{y^j} \left( 1-S\left( \frac{2\mu _1^j}{(\sigma _1^j)^2}\right) \right) ^{1-y^j} \right) \end{aligned}$$

**Figure 4:**
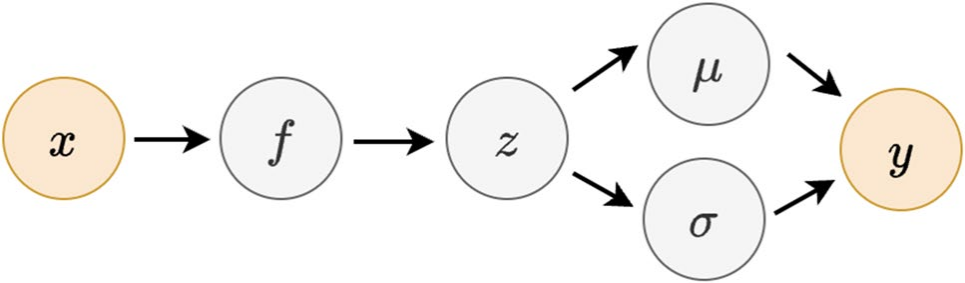
Probabilistic graph of crowdsourcing model with variational inference. For simplicity, subscripts and superscripts are excluded. Orange circles denote observed variables, whereas grey circles are unobserved. Conditional on the input *x*, a gaussian process classifier *f* produces the unobserved ground truth label *z*. For annotator *j*, conditional on the ground truth *z* (i.e., $z=0$ or $z=1$), the drift and diffusion parameters ($\mu ^j_0$ and $\sigma ^j_0$ when $z=0$, or $\mu ^j_1$ and $\sigma ^j_1$ when $z=1$) are used in a drift-diffusion model to generate the label *y*.

### Gaussian process classifier

Similar to related work in crowdsourcing^10^, a Gaussian process classifier *f* is chosen to relate inputs to ground truth labels (see Fig. 4). Inputs are denoted by $x \in \mathbb {R}^m$ so that $f: \mathbb {R}^m \xrightarrow {\phantom{0}} \mathbb {R}$ is defined with mean function $E[f(x)] = 0$ and covariance function (kernel) $K(x,x') = Cov[f(x),f(x')]$. A typical choice of covariance function is the radial basis function (i.e. $Cov(f(x),f(x')) = K_{\delta ,\gamma }(x,x') = \delta ^2 \exp {(-\gamma ||x-x'||^2})$, with |.| denoting the standard Euclidean norm. In this section, we do not define the choice of kernel. We denote $\varvec{F} = \{f_i\}_{i=1}^n$, which represents the values of the Gaussian Process at the inputs (i.e. each $f_i = f(x_i)$). Finally, a logistic function relating the gaussian process to the true outputs is assumed, i.e.,

5 $$\begin{aligned} P(z_i=1|f_i) = \frac{1}{1+e^{-f_i}} = S(f_i) \end{aligned}$$

### Variational inference

Variational inference simplifies the objective of maximizing the likelihood of the observations by maximizing the evidence lower bound (see^10,31^). Variational inference may be used to find an approximation $q(\varvec{Z},\varvec{F},\varvec{\mu }_0,\varvec{\mu }_1,\varvec{\sigma }_0,\varvec{\sigma }_1)$ of the true posterior distribution $p(\varvec{Z},\varvec{F},\varvec{\mu }_0,\varvec{\mu }_1,\varvec{\sigma }_0,\varvec{\sigma }_1|\varvec{Y})$. This is done by finding the distribution *q* that maximizes the evidence lower bound (ELBO), shown below on the right hand side of the equation below,

6 $$\begin{aligned} \ln p(\varvec{Y} \mid \theta ) \ge \mathbb {E}_{q}\left[ \ln \frac{p(\varvec{Y},\varvec{Z},\varvec{F},\varvec{\mu }_0,\varvec{\mu }_1,\varvec{\sigma }_0,\varvec{\sigma }_1)}{q(\varvec{Z},\varvec{F},\varvec{\mu }_0,\varvec{\mu }_1,\varvec{\sigma }_0,\varvec{\sigma }_1)}\right] \end{aligned}$$

### Prior distributions

Each latent variable $f_i$ is assumed to be normally distributed with mean $m_i$ and variance $v_i^2$. Uniform priors are put on the variables $\mu _0^j,\mu _1^j,\sigma _0^j$ and $\sigma _1^j$ with supports $[m_{l0},m_{u0}]$,$[m_{l1},m_{u1}]$,$[s_{l0},s_{u0}]$,$[s_{l1},s_{u1}]$ respectively, where $m_{l0},m_{u0},m_{l1},m_{u1},s_{l0},s_{u0},s_{l1},s_{u1}>0$.

#### Evidence lower bound

The mean field approximation is used, restricting the parametric form of $q(\varvec{Z},\varvec{F},\varvec{\mu }_0,\varvec{\mu }_1,\varvec{\sigma }_0,\varvec{\sigma }_1)$ (eq. 7),

7 $$\begin{aligned} \begin{array}{l} q(\varvec{Z},\varvec{F},\varvec{\mu }_0,\varvec{\mu }_1,\varvec{\sigma }_0,\varvec{\sigma }_1) = q(\varvec{Z})q(\varvec{F}) q(\varvec{\mu }_0,\varvec{\mu }_1) q(\varvec{\sigma }_0,\varvec{\sigma }_1) \\ q(\varvec{Z}) = \prod _{i=1}^{n}q_i \\ q(\varvec{F}) = \prod _{i=1}^n q(f_i) = \prod _{i=1}^n \mathbf {N}(f_i|m_i',v_i'^2) \\ q(\varvec{\mu }_0,\varvec{\mu }_1) = \prod _{i=1}^k q(\mu _0^j)q(\mu _1^j) = \prod _{i=1}^k \mathbf {U}(m'_{l0},m'_{u0}) \mathbf {U}(m'_{l1},m'_{u1}) \\ q(\varvec{\sigma }_0,\varvec{\sigma }_1) = \prod _{i=1}^k q(\sigma _0^j)q(\sigma _1^j) = \prod _{i=1}^k \mathbf {U}(s'_{l0},s'_{u0})\mathbf {U}(s'_{l1},s'_{u1}) \end{array} \end{aligned}$$

where $\mathbf{N}(f_i|m_i',v_i'^2)$ denotes the PDF of a normal distribution with mean $m_i'$ and variance $v_i'^2$ evaluated at $f_i$, and $\mathbf {U}(a,b)$ denotes a uniform distribution with support [*a*, *b*]. The ELBO may be simplified as,

8 $$\begin{aligned} \mathbb {E}_{q}\left[ \ln \frac{p(\varvec{Y},\varvec{Z},\varvec{F},\varvec{\mu }_0,\varvec{\mu }_1,\varvec{\sigma }_0,\varvec{\sigma }_1)}{q(\varvec{Z},\varvec{F},\varvec{\mu }_0,\varvec{\mu }_1,\varvec{\sigma }_0,\varvec{\sigma }_1)}\right] = \mathbb {E}_{q}\left[ \ln \frac{p(\varvec{Y}|\varvec{Z},\varvec{F},\varvec{\mu }_0,\varvec{\mu }_1,\varvec{\sigma }_0,\varvec{\sigma }_1)p(\varvec{Z}|\varvec{F})p(\varvec{F})p(\varvec{\mu }_0)p(\varvec{\mu }_1)p(\varvec{\sigma }_0)p(\varvec{\sigma }_1)}{q(\varvec{Z})q(\varvec{F})q(\varvec{\mu }_0)q(\varvec{\mu }_1)q(\varvec{\sigma }_0)q(\varvec{\sigma }_1)}\right] \end{aligned}$$

Additivity of the logarithm and linearity of the expectation yields,

9 $$\begin{aligned} \mathbb {E}_{q}\left[ \ln \frac{p(\varvec{Y},\varvec{Z},\varvec{F},\varvec{\mu }_0,\varvec{\mu }_1,\varvec{\sigma }_0,\varvec{\sigma }_1)}{q(\varvec{Z},\varvec{F},\varvec{\mu }_0,\varvec{\mu }_1,\varvec{\sigma }_0,\varvec{\sigma }_1)}\right]= & {} \mathbb {E}_{q}[\ln p(\varvec{Y}|\varvec{Z},\varvec{F},\varvec{\mu }_0,\varvec{\mu }_1,\varvec{\sigma }_0,\varvec{\sigma }_1)] +\mathbb {E}_{q}[\ln p(\varvec{Z}|\varvec{F})]\nonumber \\{} & {} \quad +\mathbb {E}_{q}[\ln p(\varvec{F})] + \mathbb {E}_{q}[\ln p(\varvec{\mu }_0)] + \mathbb {E}_{q}[\ln p(\varvec{\mu }_1)] \nonumber \\{} & {} \quad +\mathbb {E}_{q}[\ln p(\varvec{\sigma }_0)] +\mathbb {E}_{q}[\ln p(\varvec{\sigma }_1)] -\mathbb {E}_{q}[\ln q(\varvec{Z})] -\mathbb {E}_{q}[\ln q(\varvec{F})]\nonumber \\{} & {} \quad -\mathbb {E}_{q}[\ln q(\varvec{\mu }_0)] -\mathbb {E}_{q}[\ln q(\varvec{\mu }_1)] -\mathbb {E}_{q}[\ln q(\varvec{\sigma }_0)] -\mathbb {E}_{q}[\ln q(\varvec{\sigma }_1)] \end{aligned}$$

#### Objective function

By calculation of Eq. (9) (see Supplemental Information), the objective is to vary the variational parameters to maximize the objective function (i.e., the ELBO) below,

10 $$\begin{aligned}{} & {} g(\varvec{q},\varvec{F},\varvec{\mu }_0,\varvec{\mu }_1,\varvec{\sigma }_0,\varvec{\sigma }_1) \nonumber \\{} & {} \quad = \sum _{i=1}^N \ln \left[ \sum _{j=1}^k S(f_i)\left( 1-S\left( \frac{2 \mu _0^j}{\sigma _0^j}\right) \right) ^{y_i^j}\left( S\left( \frac{2 \mu _0^j}{\sigma _0^j}\right) \right) ^{1-y_i^j} +\left( 1-S(f_i)\right) \left( S\left( \frac{2 \mu _1^j}{\sigma _1^j}\right) \right) ^{y_i^j}\left( 1-S\left( \frac{2 \mu _1^j}{\sigma _1^j}\right) \right) ^{1-y_i^j}\right] \nonumber \\{} & {} \quad +\sum _{i=1}^{N}\left[ q_i \ln S(f_i) + (1-q_i) \ln (1-S(f_i))\right] - \sum _{i=1}^{N} \left[ q_i \ln q_i + (1-q_i)\ln (1-q_i)\right] \end{aligned}$$

Upon obtaining the optimal parameters from the ELBO, the distributional parameters may be estimated (see Supplemental Information),

11 $$\begin{aligned} \begin{array}{l} m_{l0} = \min \{\mu _0^j\}_{j=1}^k, \textit{ } m_{u0} = \max \{\mu _0^j\}_{j=1}^k\\ m_{l1} = \min \{\mu _0^j\}_{j=1}^k,\textit{ } m_{u1} = \min \{\mu _0^j\}_{j=1}^k\\ s_{l0} = \min \{\sigma _0^j\}_{j=1}^k,\textit{ } s_{u0} = \min \{\sigma _0^j\}_{j=1}^k\\ s_{l1} = \max \{\sigma _1^j\}_{j=1}^k,\textit{ } s_{l1} = \max \{\sigma _1^j\}_{j=1}^k\\ \end{array} \end{aligned}$$

## Experiments

### Overview of experiments

Two experiments were conducted on synthetic data in order to test the efficacy of the crowdsourcing technique. Synthetic data was generated with binary labels on a non-linear sinusoidal decision boundary (see “Generation of synthetic data” below). Reaction time data and annotations for ten hypothetical annotators were generated for the two experiments on different distributions of the annotator drift-diffusion parameters (see “Generation of annotator labels and reaction times”). Following, the crowdsourcing technique was implemented (see “Implementation of crowdsourcing technique”) with two unique cost functions (see Supplemental Information), and results were compared to a state-of-the-art technique, SVGPR^8^. Performance of the techniques were evaluated by the mean-squared errors of recovered drift-diffusion parameters, annotator sensitivity and specificity, and accuracy, sensitivity and specificity of the technique itself (see “Evaluating model performance” for more details).

### Generation of synthetic data

**Figure 5:**
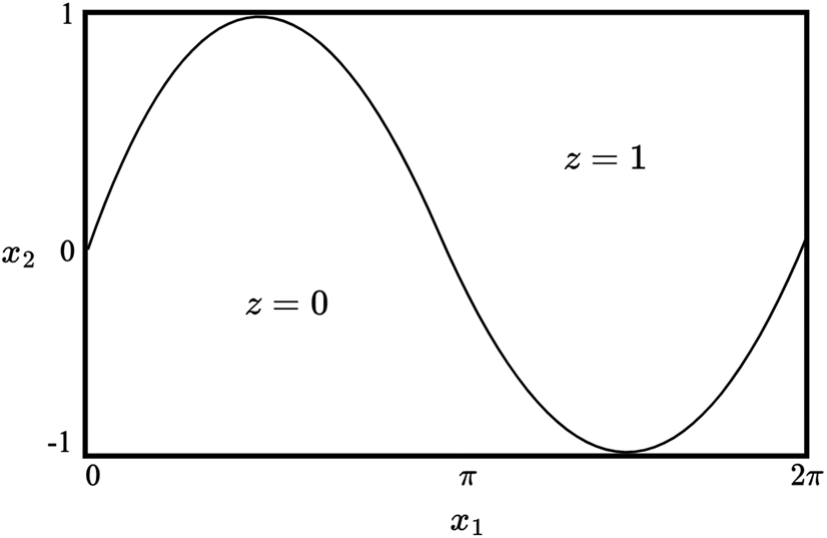
Ground truth labels for crowdsourcing task. In each experiment, two hundred inputs $\{x_i = (x_{1i},x_{2i})\}_{i=1}^{200}$ were generated from uniformly sampling on the grid $[0,2\pi ]X[-1,1]$ above. A nonlinear decision boundary, $sin(x_1)$ separates the ground truth labels $z=0$ and $z=1$, so that $z_i = 0$ if $sin(x_{1i}) < x_{2i}$, and $z_i=1$ otherwise.

In order to test the efficacy of the proposed crowdsourcing framework, two experiments were conducted on synthetic data, wherein annotator expertise and inputs varied. Corresponding to inputs, two-hundred points $\{x_i = (x_{1i},x_{2i})\}_{i=1}^{200}$ were sampled randomly (via the uniform distribution) on the grid $[0,2\pi ]X[-1,1]$ for each of the two experiments. In order to measure the technique’s ability to recover a non-linear decision boundary, ground truth labels for the inputs were assigned $z_i=0$ if $sin(x_{1i}) < x_{2i}$ and $z_i=1$ otherwise (Fig. 5) in both experiments. For every point $x_i$, a corresponding label and reaction time was generated for each annotator by sampling from their corresponding drift-diffusion model conditioned on $z_i$ defined by their drift and diffusion parameters, generated as shown below.

#### Generation of annotator labels and reaction times

For each input $x_i$, conditional on the ground truth label $z_i$, an annotator label and reaction time was generated for each annotator as follows. The process *X*(*t*) started at the origin (i.e. $X(0) = 0$) and was updated with time-step $dt = 0.01$ according to the following equation,

12 $$\begin{aligned} X(t+dt) = X(t) + \mu dt + \sigma Y(t) \end{aligned}$$

where *Y*(*t*) was drawn from a normal distribution with mean zero and variance *dt* (i.e., *Y*(*t*) represented the Wiener process), and $\mu$ and $\sigma$ represented the drift and diffusion parameters for each annotator (which was conditionally dependent on $z_i$). The process terminated at the minimum time *t* when $|X(t)| \ge 1$ (where the annotator label was set to the ground truth label $z_i$ if and only if $X(t) \ge 1$).

#### Annotators in Experiment 1

Ten annotators, defined by ground truth dependent drift and diffusion parameters, were generated (Table 1a). Conditional on $z=0$, annotator drift and diffusion parameters were drawn from uniform distributions with supports [0.1, 0.5] and [0.5, 0.9] respectively. Similarly, conditional on $z=1$, annotator drift and diffusion parameters were drawn from uniform distributions with supports [0.1, 0.5] and [0.5, 0.9] respectively. The assumption of uniformly distributed parameters in drift-diffusion models has previously been made in the literature^32^.

#### Annotators in Experiment 2

Ten annotators, defined by ground truth dependent drift and diffusion parameters, were generated (Table 1a). Conditional on $z=0$, annotator drift and diffusion parameters were drawn from uniform distributions with supports [0.1, 0.4] and [0.5, 0.7] respectively. Similarly, conditional on $z=1$, annotator drift and diffusion parameters were drawn from uniform distributions with supports [0.2, 0.5] and [0.5, 0.9].

### Implementation of crowdsourcing technique

Three methods were implemented in both experiments. Method 1 (see Supplemental Information) involved maximization of the ELBO with gradient ascent, with a regularizer term in the ELBO to enforce reaction time statistics. Method 2 (see Supplemental Information) involved maximization of the ELBO with gradient ascent, followed by rescaling of the drift and diffusion parameters based on reaction time data constraints. Method 3 involved implementation of a technique in the literature known as scalable variational Gaussian processes for crowdsourcing (SVGPCR)^8^, with uniform priors on the sensitivity and specificities of annotators. Method 3 served as a control experiment to assess the performance of methods 1 and 2 with respect to inference of ground-truth labels and annotator sensitivities and specificities.

#### Gradient ascent

The following implementation was used for all three methods across the two experiments. For maximization of the ELBO, gradient ascent was implemented in *Python* with the package *autograd* with learning rate $\alpha = 0.01$. Initial values were set to 0.5 for all parameters. All variational parameters were constrained to be between 0 and 1. This was enforced by adding the following function to the ELBO (where *v* denotes a vector of parameters, for example, $\mathbf {\mu _0}$),

13 $$\begin{aligned} c(v) = -k^{\beta (\max (v)-1)}-e^{-\beta \min (v)} \end{aligned}$$

**Figure 6:**
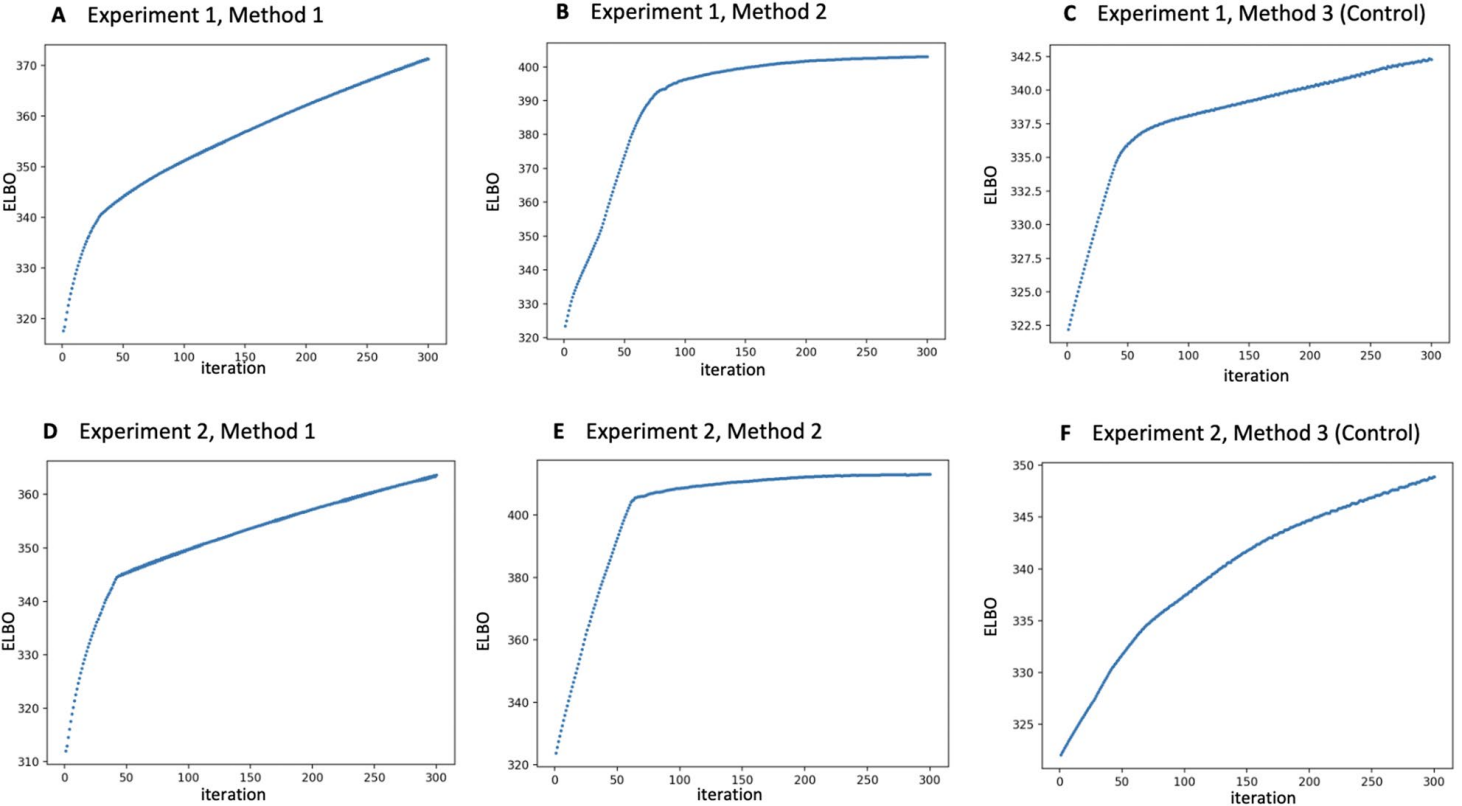
Evaluation of the ELBO from experiment 1 (**A–C**) and experiment 2 (**D–F**) during gradient ascent. The vertical axis depicts the value of the ELBO, whereas the horizontal axis depicts the iteration number in gradient ascent. The number of iterations in gradient ascent was the latter of 300 iterations, or a change in the ELBO of less than 0.1 across two subsequent iterations. In all cases, this resulted in 300 iterations of gradient ascent.

Simulations were implemented with $\beta = 30$. Gradient ascent terminated either when three hundred iterations were completed, or when the change in the ELBO across iterations was less than 0.1 (whichever came later). In all cases, this led to three hundred iterations of gradient ascent (Fig. 6).

#### Gaussian process classifier

The values of the Gaussian process ($f_i$) obtained from variational inference were used with the inputs ($x_i$) to train the Gaussian process classifier. Specifically, a Gaussian process regressor relating $x_i$ to $f_i$ was trained in Python with the package *Sklearn*^33^ implementing algorithm 2.1 in *Rasmussen*^34^. A radial basis function (RBF) summed with a white kernel was chosen as the underlying kernel of the Gaussian process. During training, the length scale bounds for the RBF kernel and white kernel respectively were constrained between .001 to 1000 .001 to 10. Upon obtaining the kernel parameters, training of the Gaussian process classifier terminated. Given a point $x^*$, with predicted-value predicted $f^*$, the label $z^* = 1$ was assigned if and only if $S(f^*) > 0.5$, i.e., $f^* > 0$.

#### Evaluating model performance

**Figure 7:**
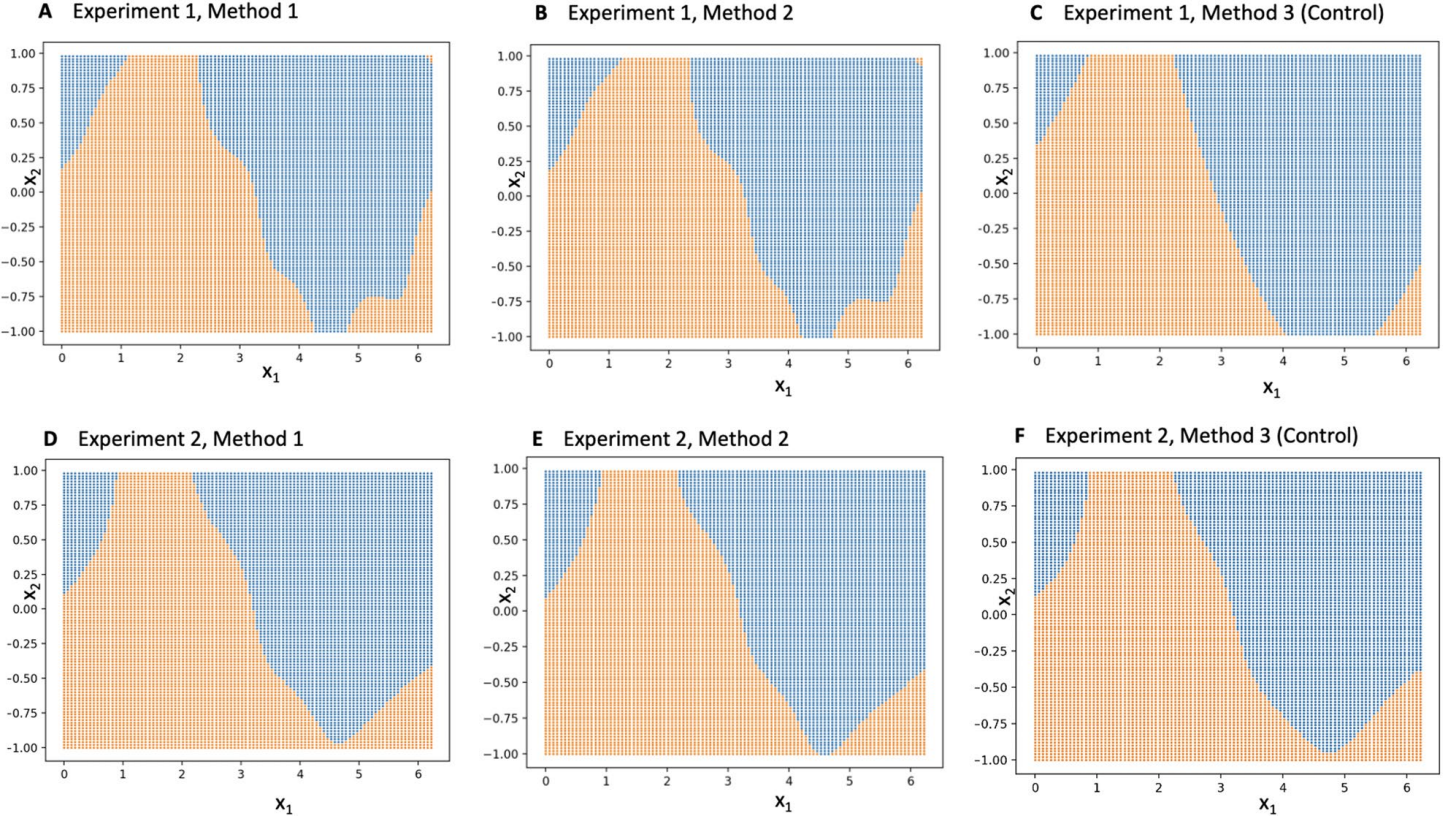
Visualization of Gaussian process classifier predictions for the three methods in experiment 1 (**A–C**) and experiment 2 (**D–F**) on the sinusoidal dataset. The horizontal axis ($x_1$) refers to the first dimension of the input, while the vertical axis ($x_2$) refers to the second dimension. Gaussian process predicted labels of 0 are depicted in orange; predicted labels of 1 are depicted in blue.

The following metrics were reported to evaluate the efficacy of the crowdsourcing framework: (1) the mean-squared error (MSE) of the recovered drift and diffusion parameters, (2) the MSE of the annotator sensitivity and specificity, (3) the overall accuracy, sensitivity and specificity of the crowdsourcing technique on the predicted labels of the inputs and (4) the accuracy, sensitivity and specificity of the crowdsourcing technique on a classification task. The classification task involved testing of the Gaussian process classifier on 10,000 points uniformly sampled on $[0,2\pi ]X[-1,1]$. These points were held constant across the three methods, but resampled for each of the two experiments. Ground-truth labels were as described in Fig. 7.

**Table 1:** (A) Summary statistics (mean, standard deviation, minimum and maximum) of annotator sensitivity and specificity for experiment one and experiment two. Each experiment involved ten annotators. (B, C) Display MSE of ground truth dependent drift and diffusion parameters, MSE of annotator sensitivity and specificity, and accuracy, sensitivity and specificity of the crowdsourcing technique and gaussian process classifier in experiments 1 and 2 respectively.

Metric	Experiment 1	Experiment 2	
(A)			
Mean annotator sensitivity	78.0	84.3	
Standard deviation of annotator sensitivity	1.16	.756	
Minimum annotator sensitivity	56.4	67.6	
Maximum annotator sensitivity	97.4	97.3	
Mean annotator specificity	78.6	79.1	
Standard deviation of annotator specificity	1.00	0.832	
Minimum annotator specificity	59.2	62.7	
Maximum annotator specificity	98.0	94.5	

Metric	Method 1	Method 2	Method 3
(B)			
MSE $\mu _0$	0.0755	0.0684	–
MSE $\mu _1$	0.126	0.0841	–
MSE $\sigma _0$	0.0903	0.114	–
MSE $\sigma _1$	0.0697	0.104	–
MSE annotator sensitivity	0.0414	0.0538	0.156
MSE annotator specificity	0.0566	0.0656	0.0702
Accuracy (crowdsourced prediction)	99.0	99.0	98.5
Sensitivity (crowdsourced prediction)	99.1	99.1	99.1
Specificity (crowdsourced prediction)	98.9	98.9	97.9
Accuracy (Gaussian process classifier)	96.2	96.1	91.2
Sensitivity (Gaussian process classifier)	97.2	97.3	87.8
Specificity (Gaussian process classifier)	95.2	94.8	94.8
(C)			
MSE $\mu _0$	0.0765	0.0296	–
MSE $\mu _1$	0.0305	0.0319	–
MSE $\sigma _0$	0.0698	0.0907	–
MSE $\sigma _1$	0.121	0.134	–
MSE annotator sensitivity	0.0427	0.0483	0.0621
MSE annotator specificity	0.0293	0.0308	0.0221
Accuracy (crowdsourced prediction)	99.0	99.0	99.0
Sensitivity (crowdsourced prediction)	98.9	98.9	98.9
Specificity (crowdsourced prediction)	99.1	99.1	99.1
Accuracy (Gaussian process classifier)	96.2	96.6	96.3
Sensitivity (Gaussian process classifier)	97.4	97.4	97.6
Specificity (Gaussian process classifier)	94.9	95.7	94.9

### Simulation results

Method 2 yielded lower MSE values (Table 1) for the drift parameters than method 1 in experiment A (0.0684 vs. 0.0755 respectively for $\mu _0$, and 0.0841 vs. 0.126 respectively for $\mu _1$). In experiment B, method 2 resulted in lower MSE for $\mu _0$ than method 1 (0.0296 vs. 0.0765 respectively), but resulted in a slightly greater MSE for $\mu _1$ (0.0319 vs. 0.0305 respectively). On the other hand, method 2 yielded higher MSE values for the diffusion parameter in both experiment A (0.114 vs. 0.0903 respectively for $\sigma _0$, and 0.126 vs. 0.0841 respectively for $\sigma _1$) and experiment B (0.0907 vs. 0.0698 respectively for $\sigma _0$, and 0.134 vs. 0.121 respectively for $\sigma _1$). For both experiments across all three methods, the maximum MSE of annotator sensitivity was 0.156 (obtained in experiment A, method 3) and the maximum MSE of annotator specificity was 0.0702 (also obtained in experiment A, method 3).

Accuracy, sensitivity and specificity on crowdsourced labels ranged between 97.9 to 99.1 percent across the two experiments and three methods. In experiment A, the highest metrics were achieved in methods 1 and 2; in experiment B, all three methods achieved the same metrics. Evaluation of the Gaussian process classifier (Fig. 6) provided slightly lower accuracies, sensitivities and specificities, which ranged between 87.8 to 97.6$\%$ across both experiments and the three methods.

## Discussion

Distributional assumptions in crowdsourcing, when made about annotators, typically focus on priors of the sensitivity and specificity of annotators (or more generally, priors on a confusion matrix of annotators), treating models of decision making that may be applied to annotators as disparate from the crowdsourcing task. This study sought to serve as a preliminary investigation into the usage of a neuroscientifically validated model of decision making to model behavior of annotators in a crowdsourcing task. Specifically, results showed that when drift-diffusion models were the generative process for annotator labels, variational inference allowed for recovery of annotator parameters with small MSE (<0.0841 for the drift parameter and <0.134 for the diffusion parameter) and similar accuracies to a state-of-the-art (SVGPR^8^) approach (accuracies were either greater or the same in method 1 and method 2 in all cases). To the authors’ knowledge, application of neuroscientifically validated models of decision making have not been previously applied to crowdsourcing tasks.

The benefit of usage of models of decision making in crowdsourcing tasks relates to the ability to learn extensively about annotators. Typically, only the reliabilities of annotators are learned in crowdsourcing tasks. Drift-diffusion modeling makes it possible to learn neurological correlates. Studies show that drift-diffusion parameters can be used to predict neural activity^15,35^. Given the integrative nature of machine learning with computational neuroscience, neural activity of accurate annotators in turn may be useful in development of machine learning algorithms, in line with literature showing the synergetic nature between the two fields^36^. An additional benefit is neurological correlates may be used as selection criter for annotators to be used in future labeling tasks, which may not be the same as the original crowdsourcing task. One study found that activity in the frontal eye fields correlate to drift-diffusion parameters in annotators^37^. Annotators ascertained to have greater frontal eye field activity may be selected in future tasks related to visual discrimination.

An interesting facet of the drift-diffusion model is the *implicit* relationship between response time and accuracy. The relationship can be seen by the following two observations: (1)The drift rate represents the average change in the process with respect to time; therefore, a larger drift rate (with the diffusion rate held constant) implies earlier termination of the process in expectation. (2) Larger drift rates imply greater accuracy. Consider an annotator annotator with drift and diffusion rate (conditional on $z=0$) given by $\mu _0$ and $\sigma _0$ respectively. The specificity of the annotator, given by $S(\frac{2\mu _0}{\sigma _0^2})$, is monotonically increasing in the drift rate $\mu _0$. Observations (1) and (2) together allude that an annotator with lower average reaction times may be deduced to have a higher drift rate, and hence accuracy, when controlling for the diffusion parameter. In practice, however, this may not always be the case. For example, an annotator who arbitrarily assigns a label to every input in a decision making task may have low average reaction time, but also low accuracy. Therefore, the assumption that annotators make decisions according to a drift-diffusion model should be validated in practical applications. It is also worth noting that the diffusion rate plays a role in the reaction times of the annotators. The optional stopping theorem relates the expected value of the drift-diffusion process at the reaction time (denoted $E[X(\tau )]$) to the reaction time $(\tau )$ and drift rate ($\mu$), i.e. $E[X(\tau )] = \mu E[\tau ]$. Because $E[X(\tau )]$ is monotonically decreasing in the diffusion rate, increasing the diffusion rate (while fixing the drift rate) corresponds to lower reaction times.

Limitations of the study are considered. Firstly, experiments relied upon the synthetic data, which relied upon the underlying assumption that annotator decisions were indeed made through the drift-diffusion model of decision making. While literature supports the drift-diffusion model as a neuroscientifically validated model of decision making^14,15,17,35^, a robust approach would be to test the performance of the technique on legitimate data, which may contain noise. For example, in the drift-diffusion model, response times are dependent solely on the decision making process. In practice, response times may not be dependent on the decision making process. For example, consider a hypothetical situation where a loss of internet connection occurs while an annotator completes an online annotation task from a personal device, such as a smart-phone or laptop. The loss of internet connection may result in a lag-period in their response, distorting the dynamics of the decision making process. The decision making process effectively incurs a discontinuity, even though the drift-diffusion model assumes a continuous-time process. It is also possible that more complex models, such as those that consider the drift and diffusion rates to be functions of time, may be warranted, which are not considered in the scope of this study. Therefore, it is of utmost significance that future work consider non-synthetic data, in order to evaluate the predictive power of the technique in practice.

Another limitation of the study is the increased computational complexity when compared to traditional crowdsourcing techniques. In this study, four parameters were introduced ($\mu _0,\mu _1,\sigma _0,\sigma _1$) per annotator into the ELBO, increasing the number of parameters per annotator by two to the traditional approach (which only considers the sensitivity and specificity, such as SVGPR). Therefore in the binary case, such as when compared to SVGPR, this technique imposes an additional 2*N* parameters, where *N* is the number of annotators. In the non-binary case, such as with *R* labels, SVGPR makes use of an *RxR* confusion matrix for each annotator, resulting in $R^2$ parameters per annotator.^8^ In our case, however, each label corresponds to a unique drift diffusion process per annotator; hence, this results in an additional *R* parameters per annotator. Therefore, in the case of *R* labels and *N* annotators, SVGPR uses $R^2N$ parameters for annotator reliability, whereas our approach uses $(R^2 + R)N$ parameters (i.e., *RN* more parameters). As the number of annotators grow, these considerations may add significantly more parameters, which may significantly increase computation time. In terms of scalability, this means that in *I* iterations of gradient ascent, *RNI* additional derivatives must be calculated in the proposed technique. These additional computations should be kept in mind when deciding in which approach to crowdsourcing to take.

It is also worth noting that the drift-diffusion model introduced in this paper assumes constant drift and diffusion parameters. More general models may assume that the drift and diffusion rates are functions of time^38,39^. Such a model would more accurately incporate changes in decision making, as well as annotator learning that might occur in a crowdsourcing task. The drift-diffusion model also treats the decision making process soley dependent on the individual, but if annotators collaborate, the model may not include interactions between annotators. The framework presented also assumes that, conditional on the ground truth, annotator decisions are independent of the ground truth, and generated from the drift-diffusion model. In practice, this may not be the case, as two distinct inputs with the same ground truth label may contain unique information involved in annotator decision making.

Futher challenges and limitations involved with the usage of drift-diffusion models in crowdsourcing tasks may be dependent on the specific crowdsourcing task. For example, it is possible that some crowdsourcing tasks may dynamically adjust in difficulty over time, introducing dependency on previous annotations of the annotator; however, the dependency of such a task on previous annotations is outside the scope of this study. Additionally, cognitive fatigue and motivational factors (i.e., the amount of value different annotators attribute to compensation for participation in crowdsourcing tasks, or perceived importance of the crowdsourcing task), may result in atypical annotator on a case-by-case basis. This factors should be kept in mind when designing crowdsourcing tasks and implementing crowdsourcing models.

While this study did not involve human participants, a number of ethical considerations may be of relevance when obtaining crowdsourcing data for the purpose of drift-diffusion modeling in future work. Due to the literature linking drift-diffusion parameters to neurological correlates and behavior, informed consent should be obtained from participants in crowdsourcing tasks wherein drift-diffusion modeling is implemented. In particular, it is suggested that participants be notified about the intended use of their data, provisions of withdrawal of their data, and details about the objectives in relation to their data. Data privacy and anonymization should be implemented wherever possible. Study protocols should be implemented in accordance to a standard framework, such as the Declaration of Helsinki^40^. Further, the authors believe that dissemination of results from crowdsourcing tasks implementing drift-diffusion modeling should protect the anonymity of individuals. This can act to ensure prevention of misuse of annotator data, such as for behavioral based marketing purposes or monetization of annotator data.

Finally, in addition to addressing ethical considerations, a comprehensive comparison of the performance of various models of decision making (such as the ballistic linear accumulator^41^ and reinforcement learning models^42^) and crowdsourcing priors in the literature should be conducted.

## Supplementary Information

Supplementary Information.

## Data Availability

All data generated in the experiments will be made publicly available on Open Science Framework (OSF) pending acceptance of the manuscript.
