# Supplementary material for: Crowdsourcing with the drift diffusion model of decision making
Source: Sci Rep. 2024 May 17;14:11311. doi: 10.1038/s41598-024-61687-y (PMC11649787; doi:10.1038/s41598-024-61687-y)
Supplement: Supplementary file 1 — Supplementary Information. [file 41598_2024_61687_MOESM1_ESM.pdf]

# Supplementary Information

**Shamal Lalvani[1,*] and Aggelos Katsaggelos[1]**

[1]Northwestern University, Department of Electrical and Computer Engineering, Evanston, 60201, USA
[*]shamal.lalvani@northwestern.edu

## Derivation of Annotator Sensitivity and Specificity

The annotator sensitivity and specificity may be derived using a simular argument from *Ross*[1] involving the optional stopping theorem for martingales. Consider annotator $j$, where $1 \leq j \leq k$. The sensitivity and specificity of annotator $j$ is given by the probability that the respective drift-diffusion process reaches the upper boundary conditional on the ground-truth labels (i.e., $z = 1$ and $z = 0$ respectively). Let $z = 1$, and let $X^j(t) = \mu_1^j + \sigma_1^j W(t)$ denote the drift-diffusion process for annotator $j$ (for simplicity, denoted $X(t) = \mu_1 + \sigma_1 W(t)$), where $W(t)$ is the Wiener-process. Let $\tau = \min\{t : |X(t)| = 1\}$ denote the stopping-time for the process. The sensitivity of the annotator is given by $\alpha = P(X(\tau) = 1)$. Note that for any real-valued $c$, $Y(t)$ defined below is a martingale[1],

$$Y(t) = e^{cW(t) - c^2 \frac{t}{2}} \tag{1}$$

By definition of $X(t)$, it follows that $W(t) = \frac{X(t) - \mu_1 t}{\sigma_1}$. Substitution of $W(t)$ into eq. 1 yields,

$$Y(t) = e^{\frac{c}{\sigma_1} X(t) - \frac{c}{\sigma_1} \mu_1 t - c^2 \frac{t}{2}} \tag{2}$$

Putting $c = \frac{-2\mu}{\sigma}$ gives,

$$Y(t) = e^{\frac{-2\mu_1}{\sigma^2} X(t)} \tag{3}$$

Note that $Y(t)$ has mean one. By the optional stopping theorem[1], $E[Y(\tau)] = 1$ so that,

$$E[e^{\frac{-2\mu_1}{\sigma^2} X(\tau)}] = 1 \tag{4}$$

Equivalently,

$$\alpha e^{\frac{-2\mu_1}{\sigma_1^2}} + (1 - \alpha) e^{\frac{2\mu_1}{\sigma_1^2}} = 1 \tag{5}$$

Let $S(a) = \frac{1}{1+e^{-a}}$ denote the logistic curve. Simplification of the equation above yields,

$$\alpha = \frac{1}{1 + e^{\frac{-2\mu_1}{\sigma_1^2}}} = S(\frac{2\mu_1}{\sigma_1^2}) \tag{6}$$

It may similarly be shown that the specificity $(\beta)$ is given by,

$$\beta = \frac{1}{1 + e^{\frac{-2\mu_0}{\sigma_0^2}}} = S(\frac{2\mu_0}{\sigma_0^2}) \tag{7}$$

## Derivation of Log-Likelihood

Note that by independence of annotators,

$$P(\mathbf{Y}_i | x_i, \boldsymbol{\mu}_0, \boldsymbol{\mu}_1, \boldsymbol{\sigma}_0, \boldsymbol{\sigma}_1) = \prod_{j=1}^{k} P(y_i^j | x_i, \mu_0^j, \mu_1^j, \sigma_0^j, \sigma_1^j) \tag{8}$$

Note also that by the law of total expectation,

$$P(y_i^j|x_i,\mu_0^j,\mu_1^j,\sigma_0^j,\sigma_1^j) = p_i P(y|z=0,x_i,\mu_0^j,\sigma_0^j) + (1-p_i)P(y|z=1,x_i,\mu_1^j,\sigma_1^j) \tag{9}$$

It follows that,

$$P(\mathbf{Y}|\mathbf{X},\boldsymbol{\mu}_0,\boldsymbol{\mu}_1,\boldsymbol{\sigma}_0,\boldsymbol{\sigma}_1) = \prod_{i=1}^{n}\prod_{j=1}^{k}[p_i P(y_i^j|z_i=0,x_i,\mu_0^j,\sigma_0^j) + (1-p_i)P(y_i^j|z_i=0,x_i,\mu_1^j,\sigma_1^j)] \tag{10}$$

By substitution of annotator sensitivities and specificities, the log-likelihood is,

$$ln\ P(\mathbf{Y}|\boldsymbol{\mu}_0,\boldsymbol{\mu}_1,\boldsymbol{\sigma}_0,\boldsymbol{\sigma}_1,\mathbf{p}) = \sum_{i=1}^{n} ln\ \left(\sum_{j=1}^{k} p_i \left(1 - S(\frac{2\mu_0^j)}{(\sigma_0^j)^2})\right)^{y_i^j} \left(S(\frac{2\mu_0^j}{((\sigma_0^j)^2})\right)^{1-y_i^j} + (1-p_i)\left(S(\frac{2\mu_1^j}{(\sigma_1^j)^2})\right)^{y^j} \left(1 - S(\frac{2\mu_1^j}{(\sigma_1^j)^2})\right)^{1-y^j}\right) \tag{11}$$

## Evidence Lower Bound

As shown in the main paper, the ELBO is given by,

$$\mathbb{E}_q[\ln \frac{p(\boldsymbol{Y},\boldsymbol{Z},\boldsymbol{F},\boldsymbol{\mu}_0,\boldsymbol{\mu}_1,\boldsymbol{\sigma}_0,\boldsymbol{\sigma}_1)}{q(\boldsymbol{Z},\boldsymbol{F},\boldsymbol{\mu}_0,\boldsymbol{\mu}_1,\boldsymbol{\sigma}_0,\boldsymbol{\sigma}_1)} = \mathbb{E}_q[\ln p(\boldsymbol{Y}|\boldsymbol{Z},\boldsymbol{F},\boldsymbol{\mu}_0,\boldsymbol{\mu}_1,\boldsymbol{\sigma}_0,\boldsymbol{\sigma}_1)] + \mathbb{E}_q[\ln p(\boldsymbol{Z}|\boldsymbol{F})] + \mathbb{E}_q[\ln p(\boldsymbol{F})] + \mathbb{E}_q[\ln p(\boldsymbol{\mu}_0)] + \mathbb{E}_q[\ln p(\boldsymbol{\mu}_1)]$$
$$+\mathbb{E}_q[\ln p(\boldsymbol{\sigma}_0)] + \mathbb{E}_q[\ln p(\boldsymbol{\sigma}_1)] - \mathbb{E}_q[\ln q(\boldsymbol{Z})] - \mathbb{E}_q[\ln q(\boldsymbol{F})] - \mathbb{E}_q[\ln q(\boldsymbol{\mu}_0)] - \mathbb{E}_q[\ln q(\boldsymbol{\mu}_1)] - \mathbb{E}_q[\ln q(\boldsymbol{\sigma}_0)] - \mathbb{E}_q[\ln q(\boldsymbol{\sigma}_1)] \tag{12}$$

Following similar approaches in other work[2], we assume $p(\mathbf{F}) = q(\mathbf{F})$, so that the ELBO simplifies as shown below,

$$\mathbb{E}_q[\ln \frac{p(\boldsymbol{Y},\boldsymbol{Z},\boldsymbol{F},\boldsymbol{\mu}_0,\boldsymbol{\mu}_1,\boldsymbol{\sigma}_0,\boldsymbol{\sigma}_1)}{q(\boldsymbol{Z},\boldsymbol{F},\boldsymbol{\mu}_0,\boldsymbol{\mu}_1,\boldsymbol{\sigma}_0,\boldsymbol{\sigma}_1)}] = \mathbb{E}_q[\ln p(\boldsymbol{Y}|\boldsymbol{Z},\boldsymbol{F},\boldsymbol{\mu}_0,\boldsymbol{\mu}_1,\boldsymbol{\sigma}_0,\boldsymbol{\sigma}_1)] + \mathbb{E}_q[\ln p(\boldsymbol{Z}|\boldsymbol{F})]\mathbb{E}_q[\ln p(\boldsymbol{\mu}_0)] + \mathbb{E}_q[\ln p(\boldsymbol{\mu}_1)]$$
$$+\mathbb{E}_q[\ln p(\boldsymbol{\sigma}_0)] + \mathbb{E}_q[\ln p(\boldsymbol{\sigma}_1)] - \mathbb{E}_q[\ln q(\boldsymbol{Z})] - \mathbb{E}_q[\ln q(\boldsymbol{\mu}_0)] - \mathbb{E}_q[\ln q(\boldsymbol{\mu}_1)] - \mathbb{E}_q[\ln q(\boldsymbol{\sigma}_0)] - \mathbb{E}_q[\ln q(\boldsymbol{\sigma}_1)] \tag{13}$$

The terms in eq. 13 are derived below in eqs. 14 - 20,

$$\mathbb{E}_q[\ln p(\boldsymbol{Y}|\boldsymbol{Z},\boldsymbol{F},\boldsymbol{\mu}_0,\boldsymbol{\mu}_1,\boldsymbol{\sigma}_0,\boldsymbol{\sigma}_1)] =$$
$$\sum_{i=1}^{n} \ln[\sum_{j=1}^{k} S(f_i\left(1 - S\left(\frac{2\mu_0^j}{\sigma_0^j}\right)\right)^{y_i^j}\left(S\left(\frac{2\mu_0^j}{\sigma_0^j}\right)\right)^{1-y_i^j} + (1-S(f_i)\left(S\left(\frac{2\mu_1^j}{\sigma_1^j}\right)\right)^{y_i^j}\left(1 - S\left(\frac{2\mu_1^j}{\sigma_1^j}\right)\right)^{1-y_i^j}] \tag{14}$$

$$\mathbb{E}_q[\ln p(\boldsymbol{Z})] = \sum_{i=1}^{N} q_i \ln S(f_i) + (1-q_i)(1 - \ln S(f_i)) \tag{15}$$

$$\mathbb{E}_q[\ln p(\boldsymbol{\mu}_0)] + \mathbb{E}_q[\ln p(\boldsymbol{\mu}_1)] =$$
$$k\int_{m'_{l0}}^{m'_{u0}}[-\ln(m_{u0} - ml0)]\frac{1}{m'_{u0}-m'_{l0}}d\mu_0 + k\int_{m'_{l1}}^{m'_{u1}}[-\ln(m_{u1} - m_{l1})]\frac{1}{m'_{u1}-m'_{l1}}d\mu_1$$
$$= -k\ln(m_{u0} - ml0) - k\ln(m_{u1} - m_{l1}) \tag{16}$$

$$\mathbb{E}_q[\ln p(\boldsymbol{\sigma}_0)] + \mathbb{E}_q[\ln p(\boldsymbol{\sigma}_1)] =$$
$$k\int_{s'_{l0}}^{s'_{u0}}[-\ln(s_{u0} - s_{l0})]\frac{1}{s'_{u0}-s'_{l0}}ds_0 + k\int_{s'_{l1}}^{s'_{u1}}[-\ln(s_{u1} - s_{l1})]\frac{1}{s'_{u1}-s'_{l1}}ds_1$$
$$= -k\ln(s_{u0} - s_{l0}) - k\ln(s_{u1} - s_{l1}) \tag{17}$$

$$\mathbb{E}_q[\ln q(\boldsymbol{Z})] = \sum_{i=1}^{N} q_i \ln q_i + (1-q_i)\ln(1-q_i) \tag{18}$$

$$\begin{aligned}&\mathbb{E}_q[\ln q(\boldsymbol{\mu}_0)] + \mathbb{E}_q[\ln q(\boldsymbol{\mu}_1)] = \\ &k\int_{m'_{l0}}^{m'_{u0}}[-\ln(m'_{u0} - m'l0)]\frac{1}{m'_{u0}-m'_{l0}}d\mu_0 + k\int_{m'_{l1}}^{m'_{u1}}[-\ln(m'_{u1} - m'_{l1})]\frac{1}{m'_{u1}-m'_{l1}}d\mu_1 \\ &= -k\ln(m'_{u0} - m'_{l0}) - k\ln(m'_{u1} - m'_{l1})\end{aligned} \tag{19}$$

$$\begin{aligned}&\mathbb{E}_q[\ln q(\boldsymbol{\sigma}_0)] + \mathbb{E}_q[\ln q(\boldsymbol{\sigma}_1)] = \\ &k\int_{s'_{l0}}^{s'_{u0}}[-\ln(s'_{u0} - s'_{l0})]\frac{1}{s'_{u0}-s'_{l0}}ds_0 + k\int_{s'_{l1}}^{s'_{u1}}[-\ln(s'_{u1} - s'_{l1})]\frac{1}{s'_{u1}-s'_{l1}}ds_1 \\ &= -k\ln(s'_{u0} - s'_{l0}) - k\ln(s'_{u1} - s'_{l1})\end{aligned} \tag{20}$$

***Variational Parameters and Gradient Descent***

By substitution of eqs. 14 - 20 into eq. 13, the objective is to vary the variational parameters to maximize the objective function below,

$$\begin{aligned}&g(\boldsymbol{q}, \boldsymbol{F}, \boldsymbol{\mu}_0, \boldsymbol{\mu}_1, \boldsymbol{\sigma}_0, \boldsymbol{\sigma}_1, v_i, m_i, v'_i, m'_i, m_{l0}, m_{u0}, s_{l0}, s_{u0}, m'_{l0}, m'_{u0}, s'_{l0}, s'_{l1}) = \\ &\textstyle\sum_{i=1}^{N}\ln[\sum_{j=1}^{k}S(f_i)\left(1 - S\left(\frac{2\mu_0^j}{\sigma_0^j}\right)\right)^{y_i^j}\left(S\left(\frac{2\mu_0^j}{\sigma_0^j}\right)\right)^{1-y_i^j} + (1-S(f_i))\left(S\left(\frac{2\mu_1^j}{\sigma_1^j}\right)\right)^{y_i^j}\left(1 - S\left(\frac{2\mu_1^j}{\sigma_1^j}\right)\right)^{1-y_i^j}] \\ &+\textstyle\sum_{i=1}^{N}[q_i\ln S(f_i) + (1-q_i)\ln(1-S(f_i))] - \sum_{i=1}^{N}[q_i\ln q_i + (1-q_i)\ln(1-q_i)] - k\ln(m_{u0} - m_{l0}) - k\ln(m_{u1} - m_{l1}) \\ &-k\ln(s_{u0} - s_{l0}) - k\ln(s_{u1} - s_{l1}) - k\ln(m'_{u0} - m'_{l0}) - k\ln(m'_{u1} - m'_{l1}) - k\ln(s'_{u0} - s'_{l0}) - k\ln(s'_{u1} - s'_{l1})\end{aligned} \tag{21}$$

For our implementation, we assume that the the distributional parameters of the drift-diffusion parameters are independent of training of the ELBO, so that the objective becomes,

$$\begin{aligned}&g(\boldsymbol{q}, \boldsymbol{F}, \boldsymbol{\mu}_0, \boldsymbol{\mu}_1, \boldsymbol{\sigma}_0, \boldsymbol{\sigma}_1) = \\ &\textstyle\sum_{i=1}^{N}\ln[\sum_{j=1}^{k}S(f_i)\left(1 - S\left(\frac{2\mu_0^j}{\sigma_0^j}\right)\right)^{y_i^j}\left(S\left(\frac{2\mu_0^j}{\sigma_0^j}\right)\right)^{1-y_i^j} + (1-S(f_i))\left(S\left(\frac{2\mu_1^j}{\sigma_1^j}\right)\right)^{y_i^j}\left(1 - S\left(\frac{2\mu_1^j}{\sigma_1^j}\right)\right)^{1-y_i^j}] \\ &+\textstyle\sum_{i=1}^{N}[q_i\ln S(f_i) + (1-q_i)\ln(1-S(f_i))] - \sum_{i=1}^{N}[q_i\ln q_i + (1-q_i)\ln(1-q_i)]\end{aligned} \tag{22}$$

With the assumption, the maximum likelihood estimates of the uniform distribution[3] are used for the distributional parameters of the drift-diffusion parameters,

$$\begin{aligned}&m_{l0} = \min\{\mu_0^j\}_{j=1}^k,\ m_{u0} = \max\{\mu_0^j\}_{j=1}^k \\ &m_{l1} = \min\{\mu_0^j\}_{j=1}^k,\ m_{u1} = \min\{\mu_0^j\}_{j=1}^k \\ &s_{l0} = \min\{\sigma_0^j\}_{j=1}^k,\ s_{u0} = \min\{\sigma_0^j\}_{j=1}^k \\ &s_{l1} = \max\{\sigma_1^j\}_{j=1}^k,\ s_{l1} = \max\{\sigma_1^j\}_{j=1}^k\end{aligned} \tag{23}$$

## Incorporating Reaction Time Data

Let $\tau^j$ be a random variable that represents the reaction time for annotator $j$. Let $\hat{\tau}_i^j$ denote the observed time for which annotator $j$ labels $x_i$. For ease of notation, let $\hat{\tau}^j = \{\hat{\tau}_i^j\}_{i=1}^N$ denote the set of observed times for which annotator $j$ labels $x_1$ through $x_N$. Two methods to incorporate information from the reaction time data to the training process are described below. In the first method, training data is used to add a regularizer to the ELBO. In the second method, no regularizer is used in the ELBO. Rescaling is implemented to the drift diffusion parameters while holding the sensitivities and specificities for the annotators obtained from the ELBO fixed.

## Method 1

Let $X(t) = \mu t + \sigma dW(t)$ be a drift diffusion process, where $W(t)$ is a Wiener-process. Let $\tau = \min\{t : |X(t)| = 1\}$. By the optional stopping theorem[1],

$$E[X_\tau] = \mu E[\tau] \tag{24}$$

Note that by definition of the expectation,

$$E[X_\tau] = p(X(\tau) = 1) - p(X(\tau) = -1) = 2p(X(\tau) = 1) - 1 \tag{25}$$

Let $abs(*)$ denote the absolute value function and let $\mathbb{I}(*)$ denote the indicator function. Define $\hat{q} = \frac{1}{N}\sum_{i=1}^{N}\mathbb{I}(q_i < 0.5)$. Note that $\hat{q}$ may be used as an empirical estimate for $P(Z = 0)$. By conditioning on $z$, the following function may be used as a regularizer in the ELBO to enforce constraints from reaction time data,

$$r(\mu_0, \mu_1, \sigma_0, \sigma_1) = \sum_{j=1}^{k} abs(E[\hat{\tau}]^j - \hat{q}(\frac{2S(\frac{2\mu_0^j}{(\sigma_0^j)^2}) - 1}{\mu_0^j}) - (1-\hat{q})(\frac{2S(\frac{2\mu_1^j}{(\sigma_1^j)^2}) - 1}{\mu_1^j})) \tag{26}$$

## Method 2

A second approach is to train the ELBO presented in eq. 22 without use of the reaction time data, and subsequently re-scale the drift diffusion parameters with the reaction time data (while keeping the annotator sensitivities and specificities predicted from the ELBO fixed). For annotator $j$, let $\mu_0^j, \mu_1^j, \sigma_0^j, \sigma_1^j$ denote the estimated values of the drift-diffusion parameters after training the ELBO without a regularizer as described in Method 1. We let $\mu_0^{j\prime}, \mu_1^{j\prime}, \sigma_0^{j\prime}, \sigma_1^{j\prime}$ denote the final estimates of the drift-diffusion parameters from Method 2. Using the stopping time theorem (eq. 24), we proceed to estimate,

$$\mu_0^{j\prime} = \frac{E[X(\tau^j)|z = 0]}{E[\tau^j|z = 0]} \tag{27}$$

In the equation above, we may approximate $E[X(\tau^j)|z = 0] = \frac{2S(\frac{2\mu_0^j}{(\sigma_0^j)^2})-1}{\mu_0^j}$ as in Method 1, and approximate $E[\tau^j|z = 0] = \frac{1}{N}\sum_{i=1}^{N}\mathbb{I}(q_i > 0.5)\hat{\tau}_i^j$, i.e.,

$$\mu_0^{j\prime} = \frac{\frac{2S(\frac{2\mu_0^j}{(\sigma_0^j)^2})-1}{\mu_0^j}}{\frac{1}{N}\sum_{i=1}^{N}\mathbb{I}(q_i > 0.5)\hat{\tau}_i^j} \tag{28}$$

Next, to maintain the same specificity as derived from the ELBO, the same value must be in the sigmoid, i.e. $2\frac{\mu_0^{j\prime}}{(\sigma_0^{j\prime})^2} = 2\frac{\mu_0^j}{(\sigma_0^j)^2}$, or equivalently,

$$\sigma_0^{j\prime} = \sigma_0^j\sqrt{\frac{\mu_0^{j\prime}}{\mu_0^j}} \tag{29}$$

Analogously, $\mu_1^{j\prime}$ and $\sigma_1^{j\prime}$ may be estimated,

$$\mu_1^{j\prime} = \frac{\frac{2S(\frac{2\mu_1^j}{(\sigma_1^j)^2})-1}{\mu_1^j}}{\frac{1}{N}\sum_{i=1}^{N}\mathbb{I}(q_i \leq 0.5)\hat{\tau}_i^j} \tag{30}$$

$$\sigma_1^{j\prime} = \sigma_1^j\sqrt{\frac{\mu_1^{j\prime}}{\mu_1^j}} \tag{31}$$

## References


**1.** Ross, S. *Stochastic Processes* (John Wiley  Sons, Inc., 1996), 2 edn.

**2.** López-Pérez, M. *et al.* Learning from crowds in digital pathology using scalable variational gaussian processes. *Sci. Reports* **11**, 11612, DOI: 10.1038/s41598-021-90821-3 (2021).

**3.** M Evans, N. H. & Peacock, B. Statistical distributions, third edition. *Meas. Sci. Technol.* **12**, 117–117, DOI: 10.1088/0957-0233/12/1/702 (2001).
